# Functional Analysis of a Dominant Negative Mutation of Interferon Regulatory Factor 5

**DOI:** 10.1371/journal.pone.0005500

**Published:** 2009-05-11

**Authors:** Long Yang, Tiejun Zhao, Xiaoliu Shi, Peyman Nakhaei, Yunling Wang, Qiang Sun, John Hiscott, Rongtuan Lin

**Affiliations:** 1 Terry Fox Molecular Oncology Group, Lady Davis Institute for Medical Research, Montreal, Quebec, Canada; 2 Department of Medicine, McGill University, Montreal, Quebec, Canada; 3 Department of Microbiology & Immunology, McGill University, Montreal, Quebec, Canada; 4 Second Xiang Ya Hospital of Central South University, Changsha, China; New York University School of Medicine, United States of America

## Abstract

**Background:**

Interferon regulatory factor (IRF) family members have been implicated as critical transcription factors that function in immune response, hematopoietic differentiation and cell growth regulation. Activation of IRF-5 results in the production of pro-inflammatory cytokines such as TNFα, IL6 and IL12p40, as well as type I interferons.

**Methodology/Principal Findings:**

In this study, we identify a G202C (position relative to translation start codon) missense-mutation transcript of IRF-5 in transformed B and T cell lines, which were either infected or non-infected by viruses, and peripheral blood from ATL or CLL patients. The mutated transcript encodes a novel protein in which the sixty-eighth amino acid, Alanine, is substituted by Proline (IRF-5P68) in the DNA binding domain of IRF-5. IRF-5P68 phenotype results in a complete loss of its DNA-binding activity and functions as a dominant negative molecule through interacting with wild type IRF-5. Co-expression of IRF-5P68 inhibits MyD88-mediated IRF-5 transactivation. Moreover, Toll-like receptor (TLR)-dependent IL6 and IL12P40 production induced by lipopolysaccharide (LPS), R837 or CpG ODN 1826 was reduced in IRF-5 (P68) expressing cells as compared to the control cells.

**Conclusion:**

IRF-5P68 acts as a dominant negative regulator that interferes with IRF-5-mediated production of pro-inflammatory cytokines. The functional characterization of the novel IRF-5 mutant in transformed B and T cell lines and in ATL and CLL patients may lead to a better understanding of the role of these transcriptional regulators in hematopoietic malignancies.

## Introduction

Biochemical, molecular biological and gene knockout studies have demonstrated that the members of the interferon regulatory factor family play important roles in pathogen response, cytokine signalling, hematopoietic differentiation, regulation of cell cycle and apoptosis (reviewed in [1], [2]. IRF-5 is involved in various activities, including activation of type I interferon and inflammatory cytokine gene expression and regulation of cell growth and apoptosis [3], [4], [5], [6]. Expression of IRF-5 has been detected in B cells and dendritic cells, and is further enhanced by type I IFN or the tumor suppressor p53 [7], [8], [9]. Two nuclear localization signals (NLS) have been identified in IRF-5, both of which are sufficient for nuclear translocation and retention in virus infected cells [8]. We have demonstrated that a CRM1-dependent nuclear export pathway is involved in the regulation of IRF-5 subcellular localization. IRF-5 possesses a functional nuclear export signal (NES) that controls dynamic shuttling between the cytoplasm and the nucleus. The NES element is dominant in unstimulated cells and results in the predominant cytoplasmic localization of IRF-5 [10].

Among the IRF family members, IRF-5 and IRF-7 share the same signaling pathway that is initiated through TLR7/8 and TLR9 [5], [11]. In response to TLR7/8 or TLR9 ligand, IRF-5 and IRF-7 are recruited to myeloid differentiation primary response gene (MyD) 88. Unlike IRF-7, which binds the death domain of MyD88, IRF-5 interacts with the intermediary domain and part of the TIR domain of MyD88 [12]. The MyD88-bound IRF-5 is activated by TNF receptor-associated factor (TRAF) 6 by an as-yet-unknown mechanism. A recent study demonstrated that IRF-5 is subject to TRAF6-mediated K63-linked ubiquitination [13]. Activated IRF-5 translocates to the nucleus, binds to the ISRE motifs in the promoter sequences and results in the production of pro-inflammatory cytokines such as TNFα, IL6 and IL12p40, as well as type I IFNs [5], [11]. *IRF-5* knockout mice showed resistance to lethal shock induced by either unmethylated DNA or lipopolysaccharide, which correlates with a marked decrease in the serum levels of proinflammatory cytokines, and thus identifies IRF-5 as a key player in the TLR-Myd88 signaling pathway [5].

Using *IRF-5* gene-targeted mice, several groups have also shown that IRF-5 plays an important role in type I IFN production in a stimulation specific and cell type dependent manner [3], [4]. The induction of type I IFNs by various TLR ligands is normal in hematopoietic cells from IRF-5 deficient mice [5]. However, Newcastle disease virus-, vesicular stomatitis virus- or herpes simplex virus type 1-infected IRF-5 deficient mice have a significant decrease in the induction levels of serum type I IFN [3], [4]. The virus-mediated type I IFN production is also partially impaired in hematopoietic cells from *IRF-5* deficient mice *in vitro* [3]. Interestingly, normal type I IFN induction was observed in mouse embryonic fibroblasts (MEFs) from IRF-5 deficient mice by these viruses [3]. Paun et al. also reported that TLR9, but not TLR3/4-mediated induction of type I IFN transcription, is dependent on IRF-5 in dendritic cells [4].

IRF family members have been implicated as critical transcription factors that function in cell growth regulation and hematopoietic differentiation [14]. The expression of IRF-5 is induced by viral infection through type I IFN signaling or by DNA damage through activated p53. As a direct p53 target gene, IRF-5 also inhibits the growth of tumor cells both *in vitro* and *in vivo* [9], [15]. IRF-5 can also inhibit B-cell lymphoma tumor growth and sensitizes p53-deficient tumors to DNA damage-induced apoptosis and cell death [6], [9], [15]. Indeed, IRF-5 deficient MEFs showed a similar phenotype to p53 deficient MEFs in DNA damage-induced apoptosis, indicating that IRF-5 is essential to the apoptotic response [3]. Barnes et al. reported that IRF-5 mediated growth inhibition is associated with a p53-independent G2-M cell cycle arrest and with the stimulation of multiple cell cycle regulatory and proapoptotic genes including *Bak*, *caspase 8*, *Bax*, and *p21* [15]. In contrast, the study from Taniguchi's group indicates that the induction of *p21* gene and the induction of cell cycle arrest normally occur in *IRF-5* deficient cells. They suggest that IRF-5 is selectively involved in apoptosis but not in cell cycle arrest [3].

In this study, we identified a G202C (position relative to start codon) missense mutation (Ala68Pro) in DNA binding domain of IRF-5 (IRF-5(P68) in tumor or transform B and T cell lines and peripheral blood from chronic lymphocytic leukemia (CLL) and adult T-cell leukemia/lymphoma (ATL) patients. The IRF-5 (P68) functions as a dominant negative molecule through interacting with wildtype IRF-5, and leading to the inhibition of IRF-5 DNA binding and transactivation activity. It will be of great interest to examine the status of IRF-5 mutation in human cancers and the role of this mutation on cell growth and apoptosis.

## Materials and Methods

### Ethics Statement

This study was approved by the Research Ethics Committee of the SMBD-Jewish General Hospital (Federalwide Assurance Number: 0796). Four patients with a diagnosis of B-CLL followed at the Jewish General Hospital of Montreal were recruited in the study after informed consent.

### Plasmid constructions

Plasmids encoding GFP-IRF-5 (variant 1), Flag-IRF-5 (variant 1), Flag-IRF-5(4D) (variant 1), Myc-IRF-5 (variant 1), IRF-3(1-133)/pGEX-4T-2, IRF-7(1-150)/pGEX-4T-2 and pRLTK were described previously [10], [16]. Human IRF-5P68 cDNA (variant 1) was amplified by RT-PCR from Namalwa B-cell line and cloned into myc pcDNA3.1/Zeo (Myc-IRF-5P68) and pEGFP-C1 (GFP-IRF-5P68). GFP-IRF-5 pMSCV puro and GFP-IRF-5P68 pmscv puro were generated by cloning the AgeI-BamHI fragment (filled in with Klenow enzyme) from GFP-IRF-5 and GFP-IRF-5P68 into the BglII site (filled in with Klenow enzyme) of the pMSCV puro vector (Clontech). IRF-5(1-130)/pGEX-4T-2 and IRF-5P68 (1-130)/pGEX-4T-2 were generated by PCR and cloned into pGEX-4T-2 vector. The IL12p40-pGL3 was kindly gifted by Dr. Keiko Ozato (NICHD, Bethesda, MD, USA).

### Generation of stable cell lines and immunoblot analysis

Infectious retroparticles encoding either IRF-5P68 fused with GFP or GFP alone were generated with 293-GP2 packaging cell (Clontech, Mountain View, CA) and concentrated retroparticles were used to gene modify RAW264.7 or human embryonic kidney (HEK)293 cells. The monoclonal cell lines were selected by 4 ug/ml of puromycine in RPMI 1640 (Wisent Inc.) supplemented with 10% fetal bovine serum, sodium pyruvate and antibiotics. To determine the expression of the transgenes, equivalent amounts of whole cell extract (20 µg) were subjected to SDS-polyacrylamide gel electrophoresis (PAGE) with 10% polyacrylamide. After electrophoresis, proteins were transferred to a Hybond transfer membrane (Amersham) in a buffer containing 30 mM Tris, 200 mM glycine, and 20% methanol for 1 h. The membrane was blocked by incubation in phosphate-buffered saline (PBS) containing 5% dried milk for 1 h and then probed with anti-GFP antibody (Roche) in 5% milk-PBS at a dilution of 1∶2000. These incubations were done at 4°C overnight or at room temperature for 1 h. After three 10-min washes with PBS, the membrane was reacted with a peroxidase-conjugated secondary goat ant-mouse antibody (Amersham Corp.) at a dilution of 1∶5000. The reaction was then vsualized with an enhanced chemiluminescence detection system as recommended by the manufacturer (Amersham).

### DNA Sequencing

Peripheral blood lymphocyte samples from ATL patients and normal donors were obtained from Dr Nazli Azimi (NCI, Bethesda, MD, USA). Total RNA was isolated from PBMCs obtained from either patients or healthy donors using the QIAGEN RNeasy MiniKit. The total RNA from T cell lines (CEM, Jukart) and B cell lines (Namalwa, Mc-KAR, BJAB and BC-3) were kindly gifted by John Hiscott's lab. Those total RNAs were then subjected to reverse transcription using SuperScript II, RNAseH^−^ reverse transcriptase (Invitrogen) according to manufacturer's instructions. The cDNAs either from reverse transcription or cDNA library were amplified by PCR. The products were then digested with *Eco* RI/*Bam* HI and cloned into pBluescript KS (+). Plasmid DNA were isolated from individual clone and subjected to sequence analysis. The primers for PCR were: 5′GATCGAATTCCTCTGCCATGAACCAGTCCA3′ and 5′GATGGATCCGACCTCGTAGATCTTGTAGG3′.

### Protein expression and purification

The glutathione *S*-transferase (GST)-IRF-3, GST-IRF-5, GST-IRF-5P68 and GST-IRF-7 fusion proteins were expressed and isolated from *E.coli* DH5α following a 3 h induction with 1 mM IPTG (Pharmacia) at 37°C. Bacterial extracts in PBS containing 1% Triton X-100 were incubated with glutathione sepharose beads (Pharmacia) for 20 min at room temperature. After washed three times with PBS, the fusion proteins were eluted with 15 mM GSH in PBS.

### Electromobility shift assay (EMSA)

Whole cell extracts were prepared 24 h after transfection with expression plasmids. Cells were washed with phosphate buffered saline (PBS) and lysed in 10 mM Tris-Cl (pH 8.0), 60 mM KCL, 1 mM EDTA, 1 mM DTT, 0.5% NP-40, 0.5 mM phenylmethylsulfonyl fluoride (PMSF), leupeptin (10 µg/ml), pepstatin (10 µg/ml), aprotinin (10 µg/ml), chymostatin (0.5 µg/ml), and 0.25 µM microcystin. Equivalent amounts of whole-cell extract (20 µg) or various amounts of recombinant proteins were assayed for IRF-3, IRF-5, IRF-5P68 and IRF-7 binding by gel shift analysis using a ^32^P-labled double-stranded oligonucleotide corresponding to the interferon stimulated response element (ISRE) region of the *ISG15* promoter (5′GATCGGAAAGGGAAACCGAAACTGAAGCC3′). The binding mixture (20 µl) contained 10 mM Tris-HCl (pH 7.5), 1 mM EDTA, 50 mM NaCl, 2 mM DTT, 5% glycerol, 0.5% NP-40, 10 µg/ml of BSA, and 62.5 µg/ml of poly(dI-dC) was added to reduce non-specific binding. After 20 min of incubation with the probe, extracts were loaded on a 5% polyacrylamide gel (60∶1 cross-link) prepared in 0.5 X TBE. After 2 h electrophoresis at 200 to 250 V, the gel was dried and exposed to Kodak film at −70°C overnight. To demonstrate the specificity of protein-DNA complex formation, either a 100-fold molar excess of unlabled oligonucleotide or anti-Flag antibody were added to the binding mixture before ^32^P-labled probe was added.

### Luciferase assay

Transfections for luciferase assay were carried out in either HEK293 cells or RAW264.7 cells. Subconfluent 293 cells were transiently co-transfected with 50 ng of pRLTK reporter (*Renilla* luciferase for internal control), 100 ng of pGL-3 reporter (firefly luciferase, experimental reporter) and IRF-5, IRF-5P68 and MyD88 by calcium phosphate co-precipitation method. RAW264.7 cells seeded on 6-well plates were transfected with 100 ng of pRLTK reporter, 200 ng of pGL-3 reporter and IRF-5, IRF-5P68 and MyD88 by using lipofectamine 2000^TM^ according to the manufacturer's instructions (Invitrogen). The reporter plasmid was IL12p40 pGL-3 reporter gene. The total amounts of DNA were kept constant by supplementation with an empty vector (pcDNA3.1). At 24 h after transfection, the reporter gene activities were measured by dual-luciferase reporter assay, according to the manufacturer's instructions (Promega).

### Analysis of protein-protein interactions

HEK293 cells were transfected with expression plasmids. Whole-cell extracts (300 µg) were prepared from transfected cells and were incubated for 1 h at 4°C with 1 µg anti-Flag (M2; Sigma-Aldrich) or anti-GFP (Roche) crosslinked to 100 µl protein A/G PLUS-Agarose (Santa Cruz Biotechnology). Precipitants were washed five times with lysis buffer and proteins were eluted by boiling the beads for 3 min in 1× SDS sample buffer. Eluted proteins or 5% of the input whole cell extracts were separated by 10% SDS-PAGE. After electrophoresis, proteins were transferred for 1 h to Hybond transfer membrances (Amersham) in a buffer containing 30 mM Tris, 200 mM glycine and 20% (vol/vol) methanol. Membranes were blocked by incubation for 1h in PBS containing 5% (wt/vol) dried milk and then were probed with anti-GFP, anti-Myc (9E10; Sigma-Aldrich) or anti-Flag, each at a concentration of 1 ug/ml. Immunocomplexes were detected with a chemiluminescence-based system (ECL; Amersham).

### RT-PCR

Total RNA ( µg), isolated from monoclonal RAW264.7 cells stably carrying GFP-IRF-5P68 or GFP using the QIAGEN RNeasy MiniKit, was subjected to reverse transcription using SuperScript II, RNAseH^−^ reverse transcriptase (Invitrogen) according to manufacturer's instructions. The primers for *IL6*, FLICE-inhibitory protein (*FLIP*) and *IKBA* were described in [17] and *GAPDH* in [18]. The primers for *IL12*p40 were: 5′- TTATGTTGTAGAGGTGGACTGG-3′ and 5′-TTTCTTTGCACCAGCCATGAGC-3′. One microliters of the obtained cDNA was then amplified by PCR using Taq polymerase (Amersham) as per manufacturer's instructions. PCR conditions were as follows for all primers: 94°C for 3 min, cycles: 94°C for 45 sec, 56°C for 45 sec, and 72°C for 1 min, 22–30 times depending on optimal product detection, 72°C for 7 min. PCR samples were then resolved in a 2% agarose gel and visualized by Ethidium Bromide staining under UV light.

### Enzyme-linked immunosorbent assay (ELISA)

Monoclonal RAW264.7 cells stably carrying GFP-IRF-5P68 or GFP were mock treated (-) or treated with LPS (Sigma-Aldrich), R837 or CpG ODN 1826 (Invivogen) for 24 h. IL6 and IL12p40 were measured by ELISA according to the manufacturer's instructions (PBL biomedical laboratories and R&D system, respectively).

## Results

### Characterization of an IRF-5 mutant

IRF-5 is predominantly expressed in lymphoid cells such as T cells, B cells, monocytes and macrophages [7], [19]. While cloning the human IRF-5 gene from a T cell cDNA library, we found a G202C missense mutation that resulted in an Ala 68 Pro mutated form of IRF-5 (referred to as IRF-5P68). To confirm the existence of this mutant, we sequenced the IRF-5 cDNA amplified from a Human T-cell leukemia virus type 1-infected T cell cDNA library and an EBV-infected B cell cDNA library, respectively. As shown in **Table 1**, the mutation was detected in both cDNA libraries and the ratio of mutation was significant. We then isolated total RNAs from a number of transformed T cell lines and B cell lines and performed sequencing. The same mutation was identified in both T cell lines (CEM and Jurkat) and B cell lines (Namalwa, Mc-KAR, BJAB and BC-3). Interestingly, both virus-infected and non-infected cell lines carried this mutation. These results raised the possibility that this mutation is associated with some malignant diseases, and unlikely derived from viral infection.

**Table 1:** Identification of Ala to Pro mutation in residue 68 of IRF-5 from cDNA library and cell lines.

Source	A68 (WT)	P68(mutation)	Mutation (%)
***cDNA library***			
HTLV-1 infected T cell line	6	9	60
EBV infected B cell line	4	8	67
***T cel*** *l*			
CEM	6	8	57
Jurkat	5	6	55
***B cell***			
Namalwa	7	7	50
Mc-KAR	13	5	28
BJAB	10	9	47
BC-3	3	6	66

IRF-5 cDNA was PCR amplified from cDNA library or RT-PCR amplified with RNA isolated from indicated cell lines and cloned into Bluescript vector. Frequency of mutation was determined by DNA sequencing from plasmid DNA isolated from each sample.

To further characterize the link between this mutation and leukemic diseases, we sequenced the RNA samples obtained from peripheral blood monocytes (PBMC) of patients suffering from adult T-cell leukemia/lymphoma (ATL) and chronic lymphocytic leukemia (CLL) and healthy donors as a control. The pathogenesis of ATL results from HTLV-1 infection, while the pathogenesis of CLL is not relevant to viral infection. As shown in **Table 2**, both ATL and CLL patients carried this mutant, but healthy donors not. This reveals that the IRF-5 mutation is probably linked to ATL or CLL.

**Table 2:** Identification of Ala to Pro mutation in residue 68 of IRF-5 from peripheral blood of ALL/CLL patients.

Source	A68 (WT)	P68(mutation)	Mutation (%)
***Control***			
1	13	0	0
2	15	0	0
3	11	0	0
4	12	0	0
5	12	0	0
***ATL***			
1	12	0	0
2	16	8	33
3	10	2	16
4	11	0	0
5	8	0	0
***CLL***			
1	9	2	11
2	3	8	72
3	8	1	11
4	10	0	0

IRF-5 cDNA was PCR amplified from peripheral blood of healthy donors or ALL/CLL patients and cloned into Bluescript vector. Frequency of mutation was determined by DNA sequencing from plasmid DNA isolated from each sample.

### IRF-5P68 does not possess DNA binding activity

**Figure 1:**
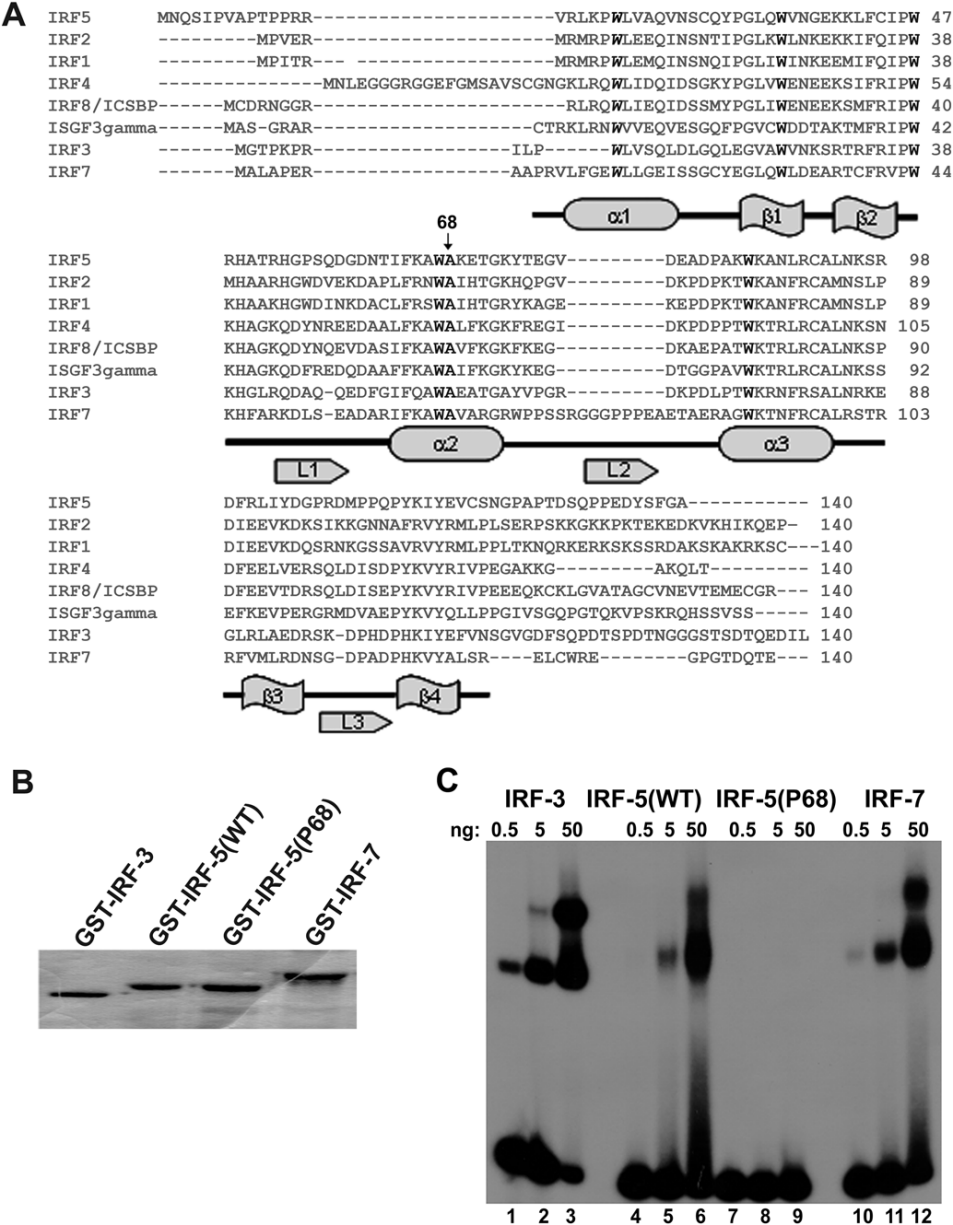
IRF-5P68 does not possess DNA binding activity. (A) Sequence alignment of the N-terminal DNA binding domain of human IRF members. (B) Coomassie staining of recombinant GST-tagged N-terminal IRF-3, IRF-5, IRF-5P68 and IRF-7. (C) EMSA of recombinant protein from a incubated with a ^32^P-labeled probe corresponding to the ISRE motif from the promoter of the gene encoding the ubiquitin-like modifier ISG15. (C) Representative green fluorescence images of living cells stably expressing IRF-5 and IRF-5P68 fused to GFP (top). The subcellular localization of the GFP-IRF-5 and GFP-IRF-5P68 was analyzed in untreated and leptomycin B-treated HEK 293 and RAW 264.7 cells. GFP fluorescence was analyzed in living cells with an Olympus BX51 fluorescence microscope using a X 40 objective. Bottom, immunoblot analysis is of GFP-IRF-5 and GFP-IRF-5P68 expression, and the expression of GFP alone as a control.

Like other IRF family members such as IRF-3 and IRF-7, IRF-5 is also characterized as a trans-activator, which contains an N-terminal DNA-binding domain and a C-terminal trans-activation domain. Given that the mutation site occurs within the N-terminal DNA-binding domain of IRF-5, we thus adopted bioinformatics analysis. As shown in **Fig. 1A**, the mutation is located in the flanking site of consensus sequence of IRF family member. We therefore hypothesized that the point mutation may ablate its DNA-binding activity. To test this hypothesis, we generated glutathione *S*-transferase (GST) tagged recombinant N-terminal DNA-binding domain of IRF-5 (referred to as GST-IRF-5), IRF-5P68 (referred to as GST-IRF-5P68), IRF-3 (referred to as GST-IRF-3) and IRF-7 (referred to as GST-IRF-7), respectively. The purity of these recombinant proteins was monitored by coomassie blue staining assay (**Fig. 1B**), and then subjected to electropheresis mobility shift assay (EMSA). The GST-IRF-3, GST-IRF-5 and GST-IRF-7 formed protein-DNA complex with the IFN-stimulated response element (ISRE) in a dose-dependent manner. In contrast, the formation of protein-DNA complex between GST-IRF-5P68 and ISRE was not detected (**Fig. 1C**). These data indicated that this missense mutation abrogates the DNA-binding activity of IRF-5P68.

It is reported that in unstimulated cells IRF-5 predominantly resides in the cytoplasm, and dynamically shuttles between nucleus and cytoplasm. To investigate whether this point mutation changes IRF-5 subcellular localization, wild type and point-mutated form of IRF-5 were linked to GFP, stably tansfected into HEK293 cells and RAW264.7 cells (mouse leukemic monocyte macrophage cell line), and examined for leptomycin B (LMB)-induced changes in subcellular localization. Like its counterpart IRF-5 wild type, the major proportion of IRF-5P68 localized in the cytoplasm of resting cells (**data not shown**). Treatment with LMB, a CRM1 inhibitor, resulted in predominant nuclear accumulation of IRF-5P68. These results suggest that this point mutation does not markedly alter IRF-5P68 protein localization in comparison with wild-type IRF-5 in resting cells.

### IRF-5P68 inhibits IRF-5-mediated transactivation activity

**Figure 2:**
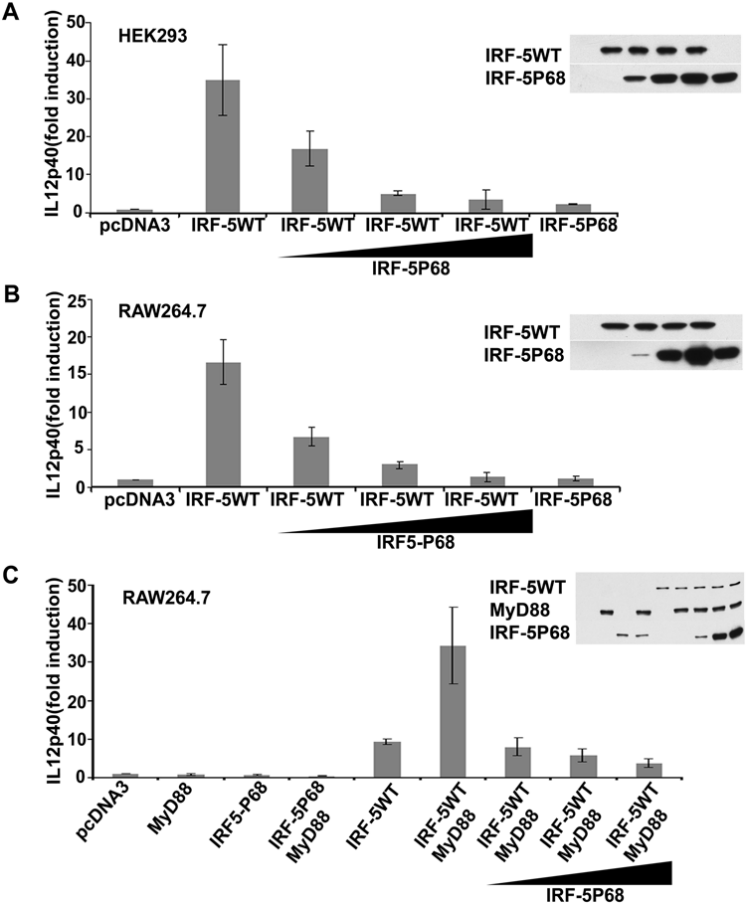
IRF-5P68 negatively regulates wild type IRF-5 transactivation activity. (A) HEK293 cells were transfected with pRLTK control plasmid (50 ng), *IL12*p40-luc reporter plasmid (100 ng) and IRF-5-expressing plasmid (200 ng) together with an increase amount of IRF-5P68 expression plasmid (0, 40, 200 and 1000 ng) as indicated. Immunoblot analysis of whole cell lysates prepared for luciferase assay was showed (right). (B) RAW264.7 cells were trasfected with pRLTK control plasmid (100ng), *IL12*p40-luc reporter plasmid (200 ng) and IRF-5-expressing plasmid (400 ng) together with an increase amount of IRF-5P68 expression plasmid (0, 80, 400 and 2000 ng) as indicated. Immunoblot analysis of whole cell lysates prepared for luciferase assay was showed (right). (C) RAW264.7 cells were trasfected with pRLTK control plasmid (100ng), *IL12*p40-luc reporter plasmid (200 ng), MyD88- and IRF-5-expressing plasmid (300 ng) together with an increase amount of IRF-5P68 expression plasmid (0, 300, 900 and 1800 ng) as indicated. Immunoblot analysis of whole cell lysates prepared for luciferase assay was showed (right). In all transfections, the pcDNA3 vector was added to bring the total plasmid to the same amount. Luciferase activity was analyzed at 24-h post-transfection by the Dual-Luciferase Reporter assay as described by the manufacturer (Promega). Relative luciferase activity was measured as-fold activation (relative to the basal level of reporter gene in the presence of pcDNA3 vector after normalization with co-transfected RLU activity); values are mean ± S.D. for three experiments.

IRF-5, as a transactivator, is a central mediator in the regulation of proinflammatory cytokines, such as TNF-α, IL6 and IL12p40. To examine the involvement of IRF-5P68 in the IRF-5-mediated transactivation activity, we measured expression of luciferase reporter gene driven by the *IL12*p40 promoter (*IL12*p40-luc) in HEK293 and RAW264.7 cells. As shown in **Fig. 2A**, in both cell lines, the IL12p40 promoter activity was effectively induced by wild type IRF-5, but not IRF-5P68. Unexpectedly, the induction of *IL12*p40 promoter activity by wild type IRF-5 was markedly decreased with the increasing amounts of IRF-5P68 expression (**Fig. 2B**). These findings suggest that IRF-5P68 might negatively regulate IRF-5 transactivation activity.

Because IRF-5 was characterized as a downstream component of TLR-MyD88- TNF receptor-associated factor (TRAF) 6 signalling, we next investigated whether the expression of IRF-5P68 interferes with MyD88-mediated IRF-5 activation. As shown in **Fig. 2C**, the *IL12*p40 promoter activity induced by MyD88 was markedly suppressed by IRF-5P68 in a dose-dependent manner. These results indicate that IRF-5P68 might participate in the modulation of TLR-signalling.

### IRF-5P68 interacts with wild-type IRF-5 and inhibited IRF-5 DNA binding activity

**Figure 3:**
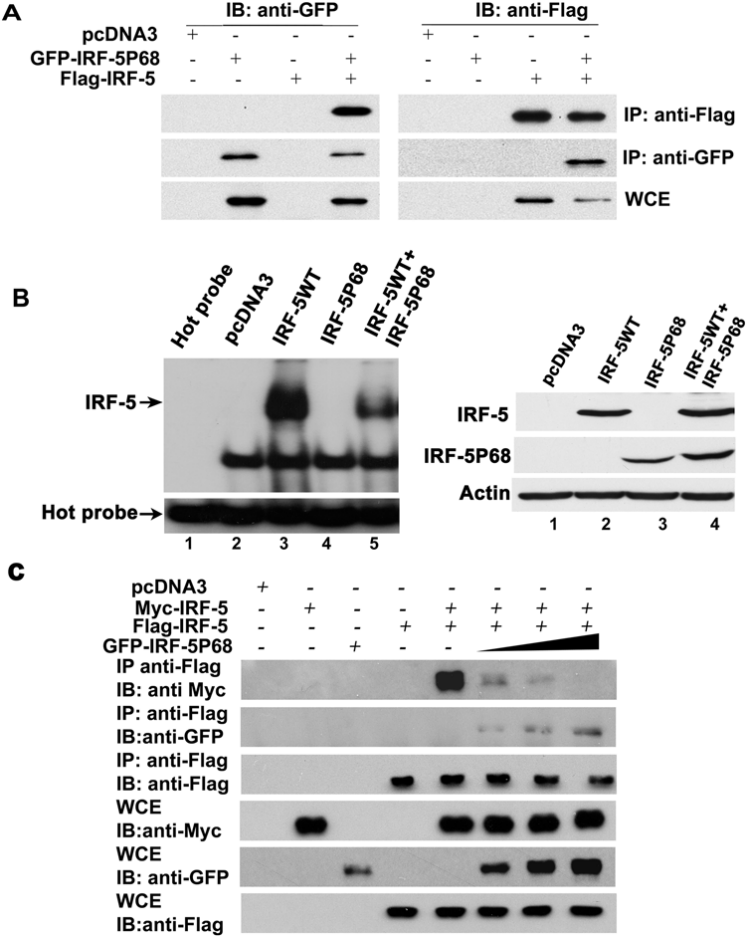
IRF-5P68 interacts with wild-type IRF-5 and inhibits IRF-5 DNA binding activity. (A) HEK293 cells were transfected with expression plasmids as indicated above the lanes. At 24 h post-transfection, Flag-tagged or GFP-tagged proteins were immunoprecipitated, separated by SDS-PAGE and probed with indicated antibodies. Whole cell extracts (WCE) were also separated SDS-PAGE and probed with antibodies as indicated. (B) HEK293 cells were transfected with expression plasmids as indicated (IRF-5WT: Flag-tagged; IRF-5P68: Myc-tagged). EMSA was performed with whole cell extracts (30 µg) derived from transfected HEK293 cells and ^32^P-labelled probe corresponds to the ISRE of the ISG-15 gene (left). Immunoblot analysis of whole cell extracts prepared for EMSA was showed (right). (C) HEK293 cells were transfected with expression plasmids as indicated above the lanes. At 24 h post-transfection, Flag-tagged proteins were immunoprecipitated, separated by SDS-PAGE and probed with indicated antibodies. Whole cell extracts (WCE) were also separated SDS-PAGE and probed with antibodies as indicated.

IRF family members such as IRF-3, IRF-5 and IRF-7 can form either homo-or hetero-dimers. Dimerization is critical for the regulation of their activity [20]. These findings suggest that the IRF-5P68 may modulate wild type IRF-5 activity through intermolecular interactions between IRF-5P68 and wild type IRF-5. To test this possibility, we expressed GFP-tagged IRF-5P68 together with Flag-tagged IRF-5 in HEK293 cells and performed coimmunoprecipitation assay. As shown in **Fig. 3A**, GFP-tagged IRF-5P68 was coimmunoprecipitated with Flag-tagged IRF-5, and vice versa, Flag-tagged IRF-5 was coimmunoprecipitated with GFP-IRF-5P68. To elucidate the possibility that binding of IRF-5P68 to wild type IRF-5 blocks IRF-5 DNA binding activity, we expressed Flag-tagged IRF-5 and Myc-tagged IRF-5P68 either alone or together into HEK293 cells, and then subjected to electrophoresis mobility-shift assay. As shown in **Fig. 3B**, Flag-tagged IRF-5 alone but not Myc-IRF-5P68 bound to ISRE DNA probe. This observation is consistent with previous data showing that GST-IRF-5 bound to ISRE whereas IRF-5P68 did not. Intriguingly, when coexpressed with Myc-IRF-5P68, the DNA-binding activity of Flag-IRF-5 was strikingly compromised. These observations indicate that IRF-5P68 can dimerize with wild type IRF-5 and inhibit IRF-5 DNA-binding activity.

Because the IRF-5 mutant can interact with wild type IRF-5, it should compete with wild-type IRF-5 for dimerization so as to interfere with the formation of IRF-5 dimers. To test this possibility, we expressed fixed amounts of the Flag-tagged IRF-5 and Myc-tagged IRF-5 together with increasing amounts of GFP-tagged IRF-5P68 into HEK293 cells and monitored IRF-5 dimerization by coimmunoprecipitation analysis. As shown in **Fig. 3C**, IRF-5P68 inhibited IRF-5 dimerization in a dose-dependent manner. These data suggest that IRF-5P68 interferes with the formation of IRF-5 homodimer.

### IRF-5P68 inhibits TLR-mediated endogenous IRF-5 target gene expression

**Figure 4:**
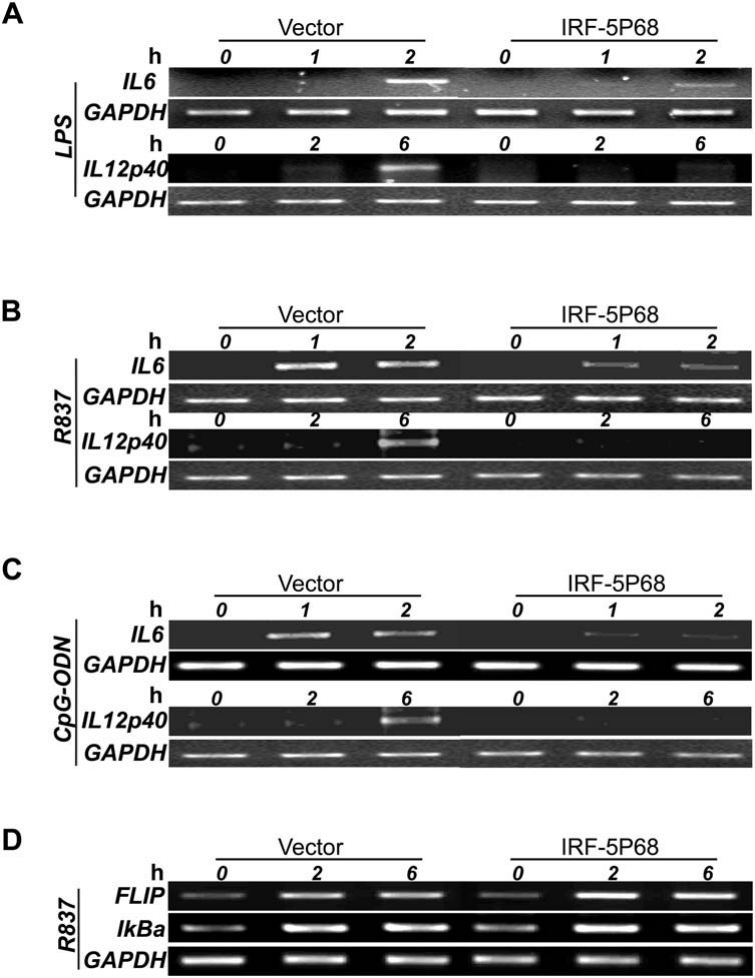
Mutated form of IRF-5 (P68) represses TLR-mediated IL6 and IL12 P40 induction. RT-PCR of transcripts encoding IL6 (*IL6*), IL12 P40 (*IL12*P40), FLIP (*FLIP*), IκBα (*IKBA*) or GAPDH (encoding glyceraldehyde phosphate dehydrogenase (loading control); *GAPDH*) in RAW264.7 cells transduced with retroviral vector encoding GFP, GFP-IRF-5(wt) or GFP-IRF-5p68 and treated (time, above lanes) with TLR4 ligand LPS (A), TLR7 ligand R837 (B and d) or TLR9 ligand ODN (C). Data are representative of three independent experiments.

To examine the physiological function of IRF-5P68 in TLR-IRF-5 signalling, we measured the TLR-induced expression of endogenous *IL12*p40 and *IL6* genes in RAW264.7 cells. To do so, we generated RAW264.7 cell lines stably expressing GFP or GFP-tagged IRF-5P68 and then stimulated the cells with TLR4 ligand LPS, TLR7 ligand R837 or TLR9 ligand CpG ODN1668. RT-PCR analysis revealed that TLR-ligand-induced expression of *IL12*p40 and *IL6* was abrogated in IRF-5P68 expressing cells (**Fig. 4A–C**), whereas, for other genes, such as *IKBA* and *FLIP*, for which IRF-5 was not required for mRNA induction, the mRNA induction levels, in response to TLR7-ligand stimuli, remained the same as those in control RAW264.7 cell lines (**Fig. 4D**). Because the latter genes depend on NF-κB transcription factor, our results revealed that the TLR-NF-κB signalling pathway was not affected by IRF-5P68, at least under our experimental conditions.

**Figure 5:**
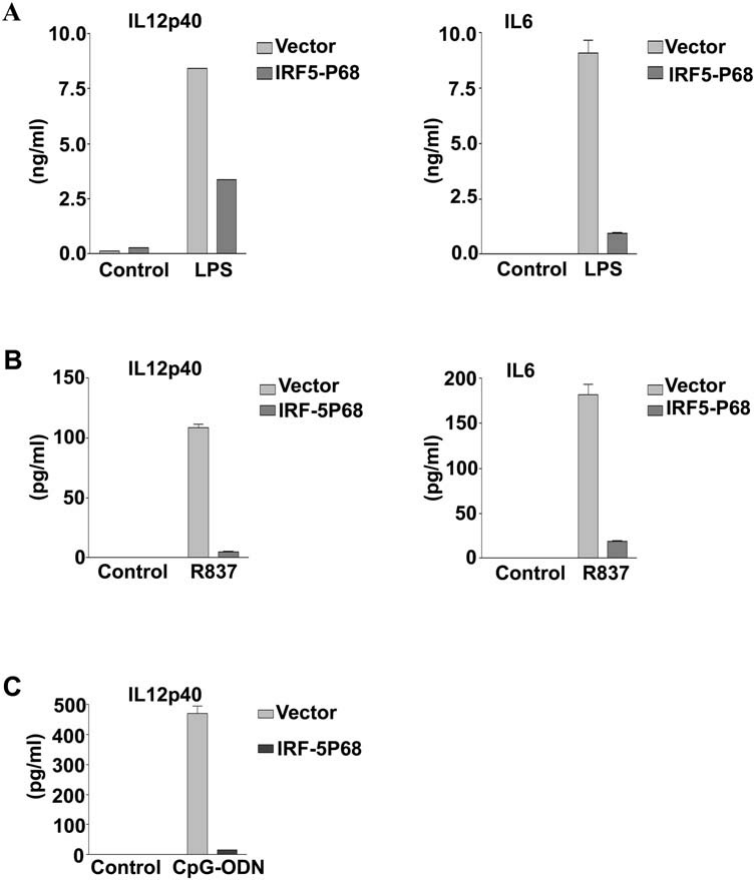
Mutated form of IRF-5 (P68) represses LPS- and R873-mediated IL6 and IL12 P40 production. ELISA of IL6 or IL12 P40 in RAW264.7 cells transduced with retroviral vector encoding GFP (vector) or GFP-IRF-5P68 (P68) and then ‘mock-stimulated’ (control) or stimulated with TLR4 ligand LPS (A), TLR7 ligand R837 (B and D) or TLR9 ligand ODN (C). Data represent the mean ± s.d. of three experiments.

We next measured TLR-ligand induced production of IL12p40 and IL6 by enzyme-linked immunosorbent assay. As shown in **Fig. 5**, the IRF-5P68 expressing RAW264.7 cells produced much less IL12p40 and IL6 in response to TLR4, TLR7 and TLR9-ligand induction than did control RAW264.7 cell lines. These results collectively demonstrate that IRF-5P68 functions as a negative regulator by specifically targeting IRF-5.

## Discussion

In this study, we have characterized a mutated form of IRF-5 in tumor or transformed B and T cell lines and in PBMCs derived from patients suffering from ATL or CLL. This is the first report linking IRF-5 mutation to certain malignant diseases, although previous studies using genetic methods have associated IRF-5 to the development of autoimmune diseases such as systemic lupus erythematosus (SLE) and rheumatoid arthritis (RA). Our study demonstrates that this mutation dominant-negatively regulates the transactivation activity of wild type IRF-5 through formation of IRF-5-IRF-5P68 heterodimers.

Recently, the crystal structure analysis of IRF-1 and IRF-2 reveal that IRF DNA-binding domain consists of three α-helices, three long loops and four stranded anti-parallel β-sheets (**Fig. 1**) [21]. Given that this structure resembles the winged helix-turn-helix (HTH)-containing DNA-binding motif and its mode of protein-DNA interaction is distinct from that of other HTH-containing proteins, the IRFs are therefore characterized as a novel HTH-containing super-family member [22]. Both IRF-1 and IRF-2 use the third α-helix to contact the major groove of the GAAA DNA sequence, and other fragments including the three loops and the α–helices to contact the adjacent DNA sequence. The secondary structure of IRF-5 DNA-binding domain is similar to that of IRF-1 and IRF-2 [21], [22], [23], suggesting that IRF-5 may utilize a similar model to recognize the similar DNA sequences. In our study, the characterized mutation site of IRF-5 is positioned in the flanking site of the fourth tryptophan within the N-terminal DNA-binding domain, which is conserved among IRF family members and is harboured by the second α-helix. The functional analysis by EMSA and luciferase assay further shows that IRF-5P68 completely loses its DNA-binding and transactivating activities. Although the structural alteration derived from the missense point mutation eradicates the DNA binding activity of IRF-5P68, it does not remarkably alter other protein properties because this mutant like its wild type predominantly localizes in the cytosol and dynamically shuttles between cytoplasm and nucleus in resting cells.

Human genome analysis shows that the mutation site of IRF-5P68 is located within Exon 3 adjacent to its 5′ end in the region of chromosome 7q32.1. Based on this information, we consulted the NCBI database for Single Nucleotide Polymorphorisms (SNPs). Unexpectedly, no SNPs in relation to this mutation site are presented in the database. We then sequenced the Exon 3 of IRF-5 gene using the genomic DNA isolated from previously described cell lines and PBMCs obtained from CLL patients carrying the mutated transcripts. Intriguingly, the mutation is undetectable at the genomic DNA level (**Table S1**), which suggests that the point mutation does not result from SNPs. To explore the molecular mechanism of this mutagenesis further, we employed the Restriction Fragment Length Polymorphorism (RFLP) method by southern-blot assay. By using Exon 3 DNA sequence as probe, a roughly 10 kb fragment was unexpectedly detected in the genomic DNA extracted from Jurkat cell line, which carries IRF-5 mutant transcripts, but not in MT4 cell line without IRF-5 mutant transcripts (**Figure S1**). In addition, the RFLP analysis by checking enzymatic cutting sites of IRF-5 pseudogene, located in chromosome 8q22.1 (pseudogene.org ID: 239282), was unable to give rise to a 10 kb fragment. These results indicate that the novel 10 kb fragment may not come from IRF-5 pseudogene but from IRF-5 gene amplification or chromosomal recombination. It will be of great interest to determine whether the IRF-5P68 mutation is associated with IRF-5 gene amplification or rearrangement. It has been demonstrated that RNA editing enzymes are also involved in mRNA point mutation [24]. The RNA editing enzymes consists of the adenosine deaminases that act on RNA (ADAR) family and the apolipoprotein B editing catalytic polypeptide (APOBEC) family [25], [26], [27], but none of them is reported to participate in G to C mRNA editing, suggesting that the IRF-5 mutation we found is unlikely caused by RNA editing.

Mutagenesis resulting in functional ablation of tumor suppressor genes is a molecular mechanism common in malignant cells to circumvent tightly controlled proliferative and survival system of mammalians. It has been well documented that the frequent loses of copies of *Rb*, especially *p53* genes in CLL contribute to disease progression, poor response to chemotherapy and poor prognostic outcome [28]. *irf-5* has been characterized as a tumor suppressor gene and the growth inhibition mediated by IRF-5 is underlined in a manner independent on *p53* [15]. IRF-5 sensitizes *p53*-deficient tumors to DNA damage-induced apoptosis and cell death [6]. In addition, DNA damaging reagents can induce IRF-5 nuclear accumulation, and *irf-5* is also associated with the stimulation of multiple cell cycle regulatory and proapoptotic genes [3]. These observations indicate that mutation of *irf-5* might be a molecular mechanism employed by malignant cells to achieve the capacity of survival or proliferation, and protecting themselves from killing by chemotherapeutical treatment. On the other hand, the discovery of an IRF-5 mutation lacking transactivation activity in ATL and CLL may unveil a clue that the transactivation activity of IRF-5 might play an essential role in its function as a turmor suppressor.

Furthermore, weak immunogenicity of CLL cells may contribute to disease progression and inhibit the effectiveness of immunotherapies [29], [30]. It has been demonstrated that in response to TLR7 activation, CLL cells increase costimulatory molecular expression, produce inflammatory cytokines and become more sensitive to killing by cytotoxic effectors [31]. Similarly, costimulation of CLL with TLR9-ligand CpG-ODN and CD40-ligand CD40LF strongly increases in inflammatory cytokine production and costimulatory molecular expression [32]. IRF-5 is a key player of TLR7/TLR9 signalling and is involved in tumor cell growth and apoptosis. The important role of IRF-5 in cell growth control is also emphasized in naïve primary B cells, in which the Epstein-Barr virus induces the negative regulators of IRF-5, the IRF-5v12 and IRF-4 [17], to neutralize TLR7 ligand-induced cell growth inhibition [33]. Thus, the mutation of IRF-5 in CLL patients may affect disease progression and immunotherapies through compromising IRF-5 transactivation activity.

In addition, recent in vivo studies utilizing mice deficient in the *Irf-5* gene have also characterized IRF-5 as a key component of death-receptor-induced apoptosis in a cell-type-specific manner [34]. IRF-5 is implicated in a stage of Fas (CD95/APO-1/TNFRSF6) signalling upstream of caspase 8 and c-jun N-terminal kinase (JNK). However, the activation of Fas receptor is not capable of inducing IRF-5 nuclear accumulation. These observations therefore raise an important issue of whether IRF-5 functions as an adaptor protein or a transcriptional activator in death-receptor-induced apoptotic signalling.

To summarize, although IRF-5P68 mutation is present in malignant hematopoietic diseases, ATL and CLL, the link between the novel IRF-5 point mutation to clinical implications, such as pathogenesis, drug resistance and prognosis, remains to be clarified. Future studies will focus on the effect of this mutation on immuno- and chemo-therapies to improve clinical diagnosis and treatment, and therefore predict the outcomes for patients suffering from hematopoietic malignant diseases such as ATL and CLL. In addition, understanding the molecular mechanism of this mutation may shed light on the development of novel tumor-suppression-gene mutation mechanisms in malignant diseases.

## Supporting Information

Table S1 Detection of Ala to Pro mutation in residue 68 of IRF-5 genome. IRF-5 exon 3 was PCR amplified from genomic DNA isolated from indicated cell lines, peripheral blood from healthy donors or CLL patients carrying IRF-5P68 transcript and cloned into Bluescript vector. The mutation of IRF-5 was determined by DNA sequencing from plasmid DNA isolated from each sample.
(0.06 MB DOC)

Figure S1 Southern bloting analysis of HindIII/XbaI-digested-genomic DNA from Jurkat (1) and MT4 (2) cell lines using the exon 3 of IRF-5 as probe.10 µg of genomic DNA isolated from either Jurkat (carring an IRF-5P68 mutation) or MT4 (without IRF-5 mutation) cell lines were digested with Hind III and Xba I, separated by 0.8% agarose gel electrophoresis, transferred to nitrocellulose membranes by upward capillary transfer in 20x saline-sodium citrate (SSC) overnight, and then hybridized to the radiolabeled whole exon 3 of human irf-5 isoform a (48% formamide, 10% dextran sulfate, 5x SSC, 1x Denhardt's solution, and 100 µg/ml salmon sperm DNA) at 42°C overnight. The membranes were washed in 2x SSC containing 0.1% SDS for 15 min at room temperature with rotation and then in 0.1% SSC containing 0.1% SDS for another 15 min at 60°C. The autoradiograms were prepared using Kodak BioMax film at −80°C with intensifying screens.
(3.52 MB TIF)
