# Supplementary material for: Functional Analysis of a Dominant Negative Mutation of Interferon Regulatory Factor 5
Source: PLoS One. 2009 May 11;4(5):e5500. doi: 10.1371/journal.pone.0005500 (PMC2677155; doi:10.1371/journal.pone.0005500)
Supplement: Table S1 — Detection of Ala to Pro mutation in residue 68 of IRF-5 genome. IRF-5 exon 3 was PCR amplified from genomic DNA isolated from indicated cell lines, peripheral blood from healthy donors or CLL patients carrying IRF-5P68 transcript and cloned into Bluescript vector. The mutation of IRF-5 was determined by DNA sequencing from plasmid DNA isolated from each sample. (0.06 MB DOC) [file pone.0005500.s001.doc]

**Table S1 Detection of Ala to Pro mutation in residue 68 of IRF-5 genome.** IRF-5 exon 3 was PCR amplified from genomic DNA isolated from indicated cell lines, peripheral blood from healthy donors or CLL patients carrying IRF-5P68 transcript and cloned into Bluescript vector. The mutation of IRF-5 was determined by DNA sequencing from plasmid DNA isolated from each sample.

| **Source(Genomic DNA)** | **A68 (WT)** | **P68 (Mutation)** | **Frequency of mutation** |
| --- | --- | --- | --- |
| ***Healthy donor blood*** | | | |
| PBMC(control) | 11 | 0 | 0% |
| ***T cell line*** | | | |
| Jurkat | 11 | 0 | 0% |
| ***B cell line*** | | | |
| BC-3 | 11 | 0 | 0% |
| ***CLL samples*** | | | |
| 003# | 12 | 0 | 0% |
| 004# | 12 | 0 | 0% |
| 005# | 12 | 0 | 0% |
