# Supplementary figures and images for: Functional Analysis of a Dominant Negative Mutation of Interferon Regulatory Factor 5
Source: PLoS One. 2009 May 11;4(5):e5500. doi: 10.1371/journal.pone.0005500 (PMC2677155; doi:10.1371/journal.pone.0005500)

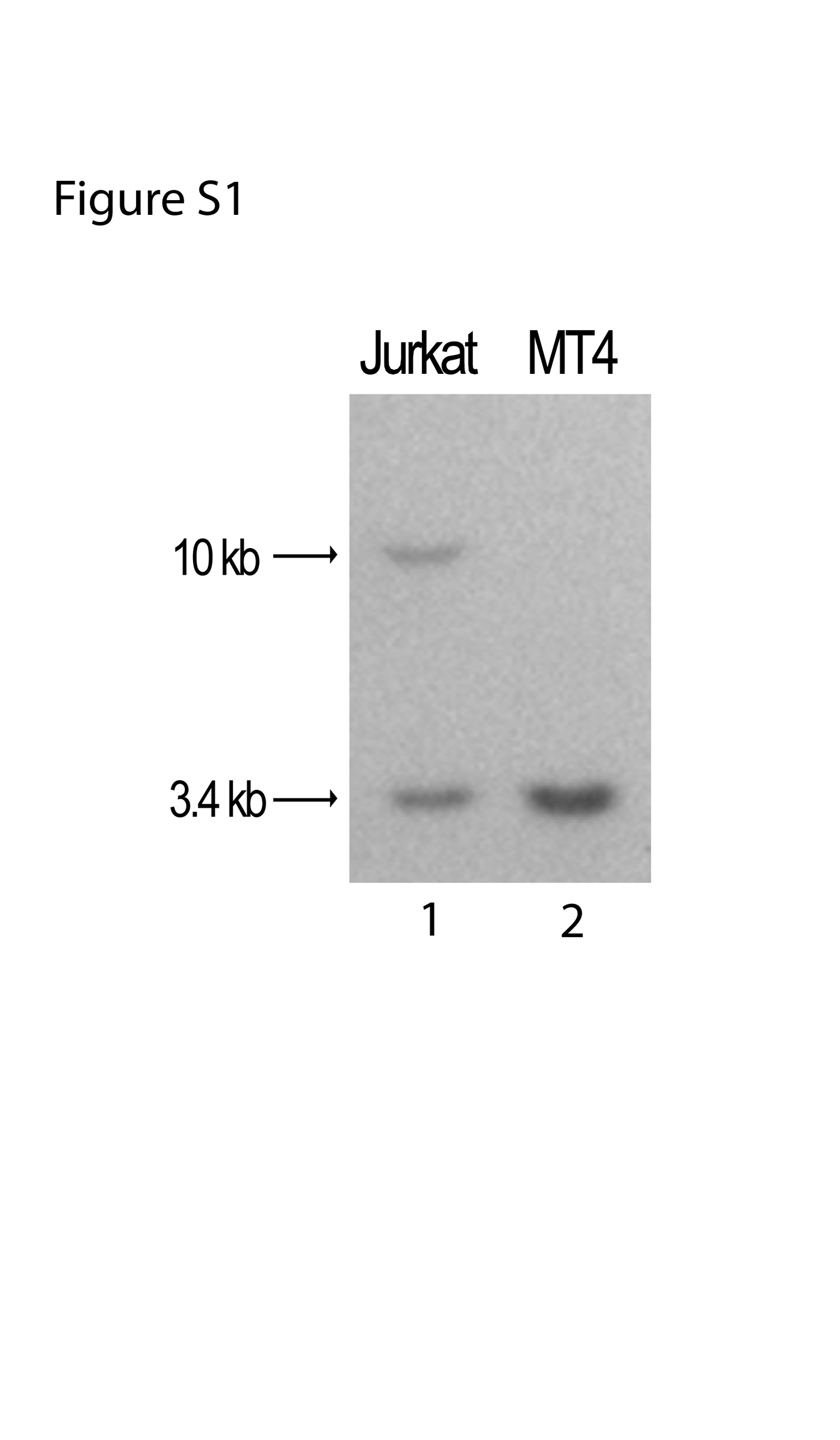

Supplement: Figure S1 — Southern bloting analysis of HindIII/XbaI-digested-genomic DNA from Jurkat (1) and MT4 (2) cell lines using the exon 3 of IRF-5 as probe.10 µg of genomic DNA isolated from either Jurkat (carring an IRF-5P68 mutation) or MT4 (without IRF-5 mutation) cell lines were digested with Hind III and Xba I, separated by 0.8% agarose gel electrophoresis, transferred to nitrocellulose membranes by upward capillary transfer in 20x saline-sodium citrate (SSC) overnight, and then hybridized to the radiolabeled whole exon 3 of human irf-5 isoform a (48% formamide, 10% dextran sulfate, 5x SSC, 1x Denhardt's solution, and 100 µg/ml salmon sperm DNA) at 42°C overnight. The membranes were washed in 2x SSC containing 0.1% SDS for 15 min at room temperature with rotation and then in 0.1% SSC containing 0.1% SDS for another 15 min at 60°C. The autoradiograms were prepared using Kodak BioMax film at −80°C with intensifying screens. (3.52 MB TIF) [file pone.0005500.s002.tif]
